# Evaluation of the effect of Modafinil in the improvement of the level of consciousness in patients with COVID‐19 encephalopathy: A randomized controlled trial

**DOI:** 10.1002/npr2.12447

**Published:** 2024-05-08

**Authors:** Fatemeh Talebi Kiasari, Mobin Naghshbandi, Maziar Emamikhah, Omid Moradi Moghaddam, Mohammad Niakan Lahiji, Mohammad Rohani, Narges Yazdi, Hamidreza Movahedi, Alireza Amanollahi, Pardis Irandoost, Roya Ghafoury

**Affiliations:** ^1^ Department of Neurology, Hazrat‐e Rasool General Hospital, School of Medicine Iran University of Medical Sciences Tehran Iran; ^2^ School of Medicine Iran University of Medical Sciences Tehran Iran; ^3^ Trauma and Injury Research Center, Critical Care Department, School of Medicine Iran University of Medical Sciences Tehran Iran; ^4^ Department of Nutrition, School of Public Health Iran University of Medical Sciences Tehran Iran

**Keywords:** COVID‐19, encephalopathy, loss of consciousness, Modafinil

## Abstract

**Aim:**

COVID‐19 can lead to encephalopathy and loss of consciousness. This double‐blinded randomized clinical trial conducted in Tehran, Iran, aimed to assess the potential effectiveness of modafinil in patients with COVID‐19‐related encephalopathy.

**Methods:**

Nineteen non‐intubated COVID‐19 patients with encephalopathy were randomized into two groups: a treatment group receiving crushed modafinil tablets and a placebo group receiving starch powder. Modafinil was administered at a dose of 100 mg every 2 h, reaching a peak dosage of 400 mg. The level of consciousness was assessed using the Glasgow Coma Score (GCS) at multiple time points on the day of medication administration. The trial was registered under IRCT20170903036041N3 on 23/5/2021.

**Results:**

The average age in the modafinil and placebo groups was 75.33 and 70 years, respectively. No significant differences were observed between the two groups in terms of chronic conditions, clinical symptoms, or laboratory data. GCS scores were similar between the groups at baseline (*p*‐value = 0.699). After four doses of modafinil, GCS scores were slightly higher in the treatment group, but this difference was not statistically significant (*p*‐value = 0.581). GCS scores after each round of drug administration didn't significantly differ between the treatment and placebo groups (*p*‐value = 0.908).

**Conclusion:**

Modafinil exhibited a slight improvement in the level of consciousness among COVID‐19 patients with encephalopathy, although this improvement did not reach statistical significance when compared to the control group. Further research with larger sample sizes and longer treatment durations is recommended to explore modafinil's potential benefits in managing altered consciousness in COVID‐19 patients.

## Abstract 2

In a double‐blinded trial in Tehran, modafinil showed potential for enhancing consciousness in COVID‐19 patients with encephalopathy but lacked statistical significance compared to placebo. Larger studies are needed to confirm modafinil's role in managing altered consciousness in COVID‐19.

## Graphical abstract

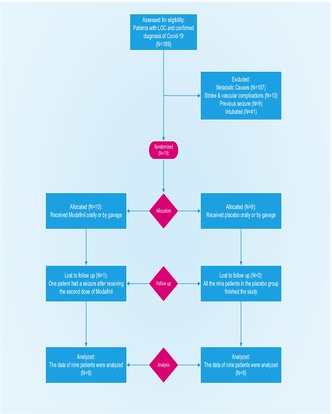

## 1 INTRODUCTION

COVID‐19 is a multisystem infection that affects individuals, both healthy individuals without underlying health conditions and those who are immunocompromised.^1^ The incubation period for COVID‐19 ranges from 4 to 14 days. Following this incubation period, various complications involving different organ systems can manifest.^1^ These complications encompass the respiratory, gastrointestinal, renal, skin, and neurological systems. Notably, neurological symptoms have been reported in 36.4% of COVID‐19 patients^2^ including symptoms such as headaches, delirium, memory issues, anosmia, dysgeusia, stroke, seizures, Guillain‐Barre syndrome, encephalopathy, encephalitis, myelitis, and loss of consciousness (LOC).^2^, ^3^

Neurologists are confronted with the challenge of addressing LOC in COVID‐19 patients. In certain instances, LOC cannot be attributed to respiratory, metabolic, or other systemic disorders and may likely be a result of pro‐inflammatory mechanisms leading to the release of cytokines within the central nervous system (CNS).^4^, ^5^ In cases of LOC‐related disorders, various CNS‐stimulating drugs, benzodiazepine receptor antagonists, or opioid receptor antagonists have been employed. Some of these treatments have resulted in improvements in alertness, attention, and neurocognitive function.^6^, ^7^ One such drug is modafinil, a non‐amphetamine CNS stimulant,^8^ approved by the FDA for the treatment of narcolepsy and obstructive sleep apnea (OSA).^9^ While the exact mechanism of action of modafinil remains incompletely understood, its effects on brain neurotransmitters are considered a plausible mechanism. Modafinil is known to elevate levels of dopamine, norepinephrine, and synaptic glutamate in regions such as the thalamus, hypothalamus, striatum, and hippocampus. Additionally, modafinil leads to a reduction in cortical gamma‐aminobutyric Acid (GABA) levels and an indirect increase in histamine in brain areas responsible for wakefulness.^10^, ^11^, ^12^

To date, no effective treatment has been proposed for LOC induced by COVID‐19. Given the aforementioned information, it is imperative to seek out a medication that can ameliorate the symptoms of decreased consciousness in these patients and potentially prevent further neurological damage. Consequently, the current study aims to investigate the effects of modafinil in improving the level of consciousness in patients diagnosed with LOC attributed to COVID‐19.

## 2 MATERIALS AND METHODS

### 2.1 Study population and eligibility criteria

This study was designed as a double‐blinded randomized clinical trial and was conducted at Rasoul Akram Hospital in Tehran, Iran, under the supervision of the Iran University of Medical Sciences. The study focused on patients who had a confirmed diagnosis of COVID‐19 and exhibited LOC resulting from COVID‐19 encephalopathy. The inclusion criteria encompassed patients who were hospitalized and diagnosed with COVID‐19 through laboratory diagnostic tests (PCR from a nasopharyngeal swab). These patients exhibited a level of impaired consciousness that was significant but did not necessitate intubation. Importantly, this decreased level of consciousness could not be explained by hypoxemia or other metabolic or systemic disorders.

Exclusion criteria were as follows: individuals without a confirmed diagnosis of COVID‐19 or those lacking symptoms of LOC, patients with a history of seizures linked to modafinil, individuals who experienced complications due to drug administration, or those whose clinical symptoms worsened, preventing the continuation of drug administration.

### 2.2 Ethics and consent

Following approval from the Ethics Committee of Iran University of Medical Sciences, this study was conducted with a commitment to ensuring the confidentiality and anonymity of all collected information. The individuals involved in the project adhered strictly to the principles outlined in the Helsinki Declaration.

Before commencing the study, a comprehensive explanation of the research objectives and methodologies was provided to the authorities at the participating centers, as well as to all research participants, in both written and verbal formats. The ethics code regarding the present study is as follows: IR.IUMS.REC.1399,1056.

The trial registration number (TRN) for this study is as follows: IRCT20170903036041N3 which was registered on 23/5/2021.

### 2.3 Intervention and outcomes

Between the winter of 2020 and the fall of 2022, a total of 189 non‐intubated patients who presented with LOC and confirmed diagnoses of COVID‐19 were initially screened at Rasoul Akram Hospital in Tehran. After a thorough evaluation, 170 patients were subsequently excluded from the study due to the presence of underlying causes for their loss of consciousness unrelated to COVID‐19. Ultimately, 19 patients were identified and enrolled in the study, each of whom had a confirmed diagnosis of COVID‐19 accompanied by a decline in their level of consciousness attributed to COVID‐related encephalopathy. Importantly, none of these patients required intubation.

**FIGURE 1:**
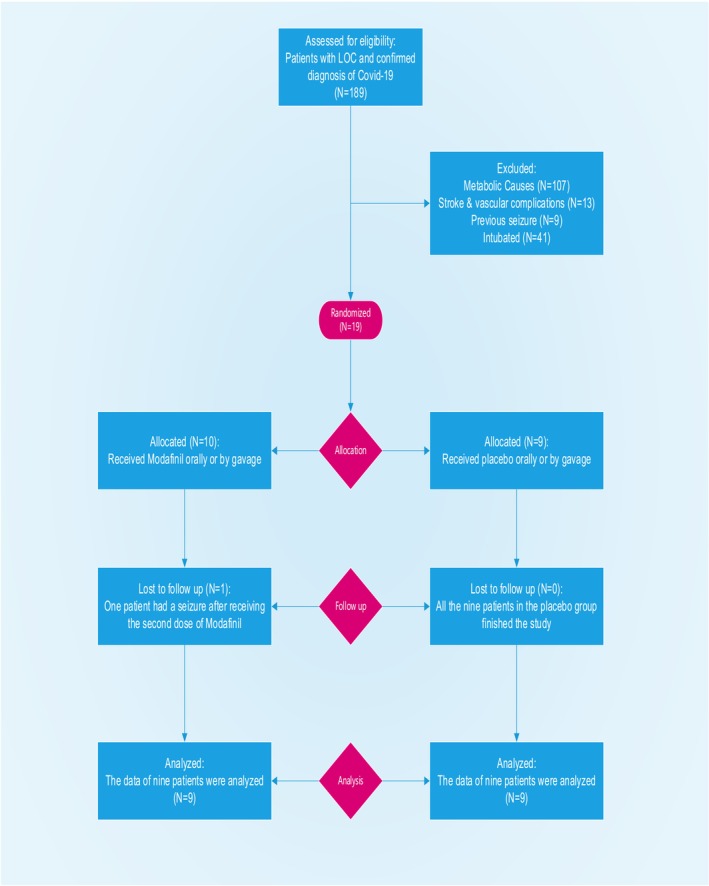
The CONSORT 2010 flowchart of the patients.

During the study, one patient experienced a seizure following the administration of the second dose of modafinil and was consequently excluded from further participation. The patient flow throughout the study is visually depicted in Figure 1. Participants were randomly assigned to either the modafinil group or the placebo group using block randomization with a 1:1 allocation ratio. This method ensured equal distribution of participants and minimized bias. Blocks of varying sizes were used to prevent predictability, and a computer‐generated randomization sequence was managed by an independent statistician.

To maintain the integrity of our trial's double‐blind design, several measures were implemented. Firstly, both the modafinil and placebo capsules were manufactured to be indistinguishable in appearance, taste, and packaging, verified through a pre‐study test with individuals not involved in the research. Secondly, the allocation sequence was securely stored and encrypted by an independent statistician, with only coded labels provided to the research team for treatment dispensation. This ensured that neither the researchers nor the participants were aware of the treatment allocations. Thirdly, outcome assessors, responsible for evaluating participants' GCS scores and other clinical outcomes, were also blinded to group allocations and trained to follow a standardized protocol for outcome evaluation, ensuring consistency and objectivity.

In the treatment group, modafinil was administered either orally or via gavage at a dose of 100 mg, commencing at 8:00 am. Subsequently, if there were no observed side effects or alterations in hemodynamics, an additional 100 mg dose was administered every 2 h, culminating in a total daily dose of 400 mg (peak plasma drug concentration is typically reached 2–4 h post‐administration).^9^ The assessment of the primary outcome, which was the level of consciousness, involved evaluations conducted before the initial drug administration at 8 am, 2 h following each dose, before the administration of the subsequent dose, and 2 h after the last dose (at hours 10, 12, 14, and 16). If the level of consciousness improved by one unit according to the Glasgow Coma Scale (GCS), the administration of the drug was continued for an additional 2 weeks at a daily dose of 400 mg. Conversely, if there was no change in the level of consciousness as per the GCS assessment, the drug administration was discontinued. In the placebo group, patients were provided with an oral placebo using the same dosing schedule. The placebo consisted of starch powder enclosed in capsule form.

The primary outcome, namely the level of consciousness, was recorded by a physician who remained unaware of the administered medication. These assessments were conducted at hours 10, 12, 14, and 16 on the day of medication administration, for both the treatment and placebo groups.

The secondary outcomes encompassed the trajectory of changes in laboratory parameters, the duration of hospitalization following drug administration (in days), and patient outcomes, including either recovery or discharge, and in some cases, unfortunately, death. These parameters were compared between the treatment and placebo groups. Additionally, any potential side effects of the drug were meticulously documented and compared between the treatment and placebo groups.

### 2.4 Data collection

Patient data were collected systematically through the use of a comprehensive checklist that encompassed demographic, laboratory, and clinical information, which included the following sections:

1. Demographic Information:
  - Age
  - Sex
  - Underlying medical conditions
  - Date of onset of clinical symptoms
  - Date of hospitalization
  - Inpatient department
2. Clinical Symptoms at the Onset of Hospitalization:
  - Detailed presentation of the patient's clinical symptoms upon admission
3. Blood Laboratory Findings:
  - Blood urea nitrogen (BUN) levels
  - Creatinine levels
  - White blood cell count (WBC)
  - Lymphocyte count
  - Neutrophil count
  - Platelet count (PLT)
  - Aspartate aminotransferase (AST) levels
  - Alanine transaminase (ALT) levels
  - Lactate dehydrogenase (LDH) levels
  - Creatine phosphokinase (CPK) levels
  - Hemoglobin (Hb) levels

This comprehensive data collection allowed for a thorough evaluation of patient characteristics and laboratory findings to be used in the analysis of treatment outcomes.

### 2.5 Statistical analysis

The data were analyzed using SPSS software version 26. The results for quantitative variables were expressed as mean and standard deviation (mean ± SD) and for categorical qualitative variables as percentages. Our analysis predominantly utilized the independent samples *t*‐test and the Mann–Whitney U test to compare baseline characteristics and primary outcomes between the modafinil and placebo groups. The *t*‐test was chosen to compare means between two independent groups when the data followed a normal distribution. Conversely, the Mann–Whitney U test was employed for non‐normally distributed data, providing a non‐parametric alternative for comparing medians. We chose the *t*‐test and Mann–Whitney U test for their established reliability in clinical trial analysis and flexibility in handling both normally and non‐normally distributed data. Our decision was informed by preliminary analysis indicating variable distributions and supported by normality tests like the Shapiro–Wilk test. The mixed linear model test was used to compare the average changes of the measured parameters over time in the two mentioned groups. The mixed linear model test was chosen because it effectively analyzes changes over time in clinical trials by accounting for within‐subject correlations, individual variations, and incomplete follow‐up data. It provides a flexible framework for modeling complex data structures and offers insight into treatment effects over time while maximizing statistical power and efficiency.

We established a significance threshold of *p* < 0.05 for all analyses. This standard threshold was selected to balance the risks of Type I and Type II errors, considering the exploratory nature of our study in a relatively new research field.

## 3 RESULTS

During 2 years, a total of 19 patients who suffered from COVID‐19‐associated encephalopathy, resulting in altered consciousness but not requiring intubation, were enrolled in this study. Randomization led to 10 patients receiving modafinil treatment and 9 patients receiving a placebo. Subsequently, one patient from the modafinil group was withdrawn due to modafinil‐induced seizures. The study continued with 9 patients in each group, and all 18 patients completed the trial, with their data being subjected to analysis.

Table 1 provides a more comprehensive breakdown of the basic characteristics of both patient groups. The randomization process ensured the absence of bias at enrollment, as no *p*‐values exceeding 0.05 were observed among various variables within the two groups. In the modafinil group, the mean age was 75.33 years (SD: 13.28), while in the placebo group, it was 70 years (SD: 12.06). Gender distribution was relatively even, with three females in the modafinil group and the remainder in the placebo group (*p*‐value = 0.347). Furthermore, there were no noteworthy disparities between the treatment and placebo groups regarding chronic conditions, clinical symptoms, or laboratory data. GCS scores were obtained and exhibited similarity between the two groups (*p*‐value = 0.699).

**TABLE 1:** Basic characteristics of the patients enrolled in the study.

Characteristics (normal ranges)	Modafinil (*n* = 9)	Placebo (*n* = 9)	*p*‐Value
Age, (years)	75.33 ± 13.28	70 ± 12.06	0.385
Gender, (F/M, %)	3/6, 50%	6/3, 200%	0.347
HTN, *n* (%)	4, 44.4%	5, 55.6%	0.99<
DM, *n* (%)	4, 44.4%	6, 66.7%	0.637
CVA, *n* (%)	2, 22.2%	1, 11.1%	0.99<
IHD, *n* (%)	1, 11.1%	2, 22.2%	0.99<
CKD, *n* (%)	2, 22.2%	0, 0%	0.471
Gradual LOC, *n* (%)	5, (55.6%)	7 (77.8%)	0.620
Dyspnea, *n* (%)	6, (66.7%)	5 (55.6%)	0.99<
Weakness, *n* (%)	6, (66.7%)	5 (55.6%)	0.99<
Cough, *n* (%)	2, (22.2%)	2 (22.2%)	0.712
Fever, *n* (%)	1, (11.1%)	1 (11.1%)	0.99<
Myalgia, *n* (%)	0, (0%)	1 (11.1%)	0.99<
Symptoms onset to hospitalization, (day)	2.78 ± 1.85	3 ± 2.73	0.843
Hospitalization to medication, (day)	5.56 ± 7.41	4.11 ± 4.59	0.626
ICU, *n* (%)	7, (77.8%)	6, (66.7%)	0.99<
Ward, *n* (%)	2, (22.2%)	3, (33.3%)	0.99<
GCS prior to medication, (3–15)	11.89 ± 1.05	11.67 ± 1.32	0.699
WBC, (4500‐11 000/mL)	6944.44 ± 4008.46	10677.78 ± 3541.46	0.059
Neutrophil, (2500‐8000/mL)	5463.56 ± 3091.67	8653.33 ± 3317.94	0.062
Lymphocyte, (800‐5000/mL)	1127.11 ± 658.88	1499.44 ± 664.97	0.250
Hemoglobin, (12.95–16.15 g/dL)	11.53 ± 2.74	11.80 ± 2.24	0.824
Platelet, (150 000‐40 000/mL)	174333.33 ± 105242.57	158333.33 ± 56222.77	0.693
BUN, (7–20 mg/dL)	33.00 ± 16.54	21.22 ± 10.03	0.085
Creatinine, (0.65–1.2 mg/dL)	2.14 ± 2.27	1.20 ± 0.27	0.234
AST, (8–33 U/L)	64.89 ± 37.19	53.67 ± 20.40	0.442
ALT, (4–36 U/L)	30.78 ± 12.03	32.67 ± 13.50	0.758
LDH, (105–333 IU/L)	683.44 ± 295.48	680.00 ± 120.85	0.975
CPK, (10–120 μg/L)	319.56 ± 360.06	182.11 ± 228.57	0.348

Abbreviations: ALT, Alanine transaminase; AST, Aspartate aminotransferase; BUN, blood urea nitrogen; CKD, chronic kidney disease; CPK, Creatine phosphokinase; CVA, cerebrovascular accident; DM, diabetes mellitus; GCS, Glasgow Coma Scale; HTN, hypertension; ICU, intensive care unit; IHD, ischemic heart disease; LDH, Lactate dehydrogenase; LOS, loss of consciousness; WBC, white blood cells.

**FIGURE 2:**
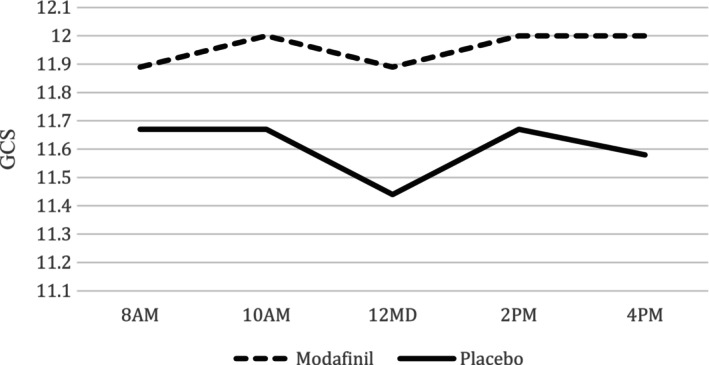
The course of GCS changes in both modafinil and placebo groups before and after receiving the drug.

Following the administration of four doses of modafinil in the treatment group and placebo in the control group, given over two‐hour intervals, GCS scores were assessed. Although the GCS score in the treatment group was marginally higher after four doses of modafinil, this difference was not statistically significant (*p*‐value = 0.581). Additionally, GCS scores after each round of drug administration did not exhibit a substantial distinction between the treatment and placebo groups. Blood samples were collected immediately after drug administration, as well as one and 3 days later, with alterations in laboratory data detailed in Table 2. No significant differences were observed between the two groups. Of note, four out of 9 patients in the treatment group (44.4%) and three patients in the placebo group (33.3%) unfortunately succumbed to their condition. However, the disparity in outcomes (discharge or death) between the two groups did not achieve statistical significance. The trajectory of GCS score changes in both groups is depicted in Figure 2.

**TABLE 2:** Alterations in GCS and laboratory parameters of the two groups.

Characteristics (normal ranges)	Modafinil (*n* = 9)	Placebo (*n* = 9)	*p*‐Value
GCS, (3–15)			
10 am	12.00 ± 1.11	11.67 ± 1.32	0.572
12 am	11.89 ± 1.45	11.44 ± 1.50	0.533
2 pm	12.00 ± 1.41	11.67 ± 1.32	0.613
4 pm	12.00 ± 1.22	11.56 ± 1.33	0.472
Mean	11.97 ± 1.25	11.58 ± 1.31	0.908
WBC, (4500–11 000/mL)			
Day 0	8077.78 ± 4276.32	9887.50 ± 4659.38	0.420
Day 1	9111.11 ± 3972.54	9612.50 ± 4605.41	0.813
Day 3	9750.00 ± 3413.81	9612.50 ± 3675.18	0.380
Mean	8950.00 ± 3562.22	9704.16 ± 3642.92	0.307
Neutrophil, (2500–8000/mL)			
Day 0	6740.00 ± 4174.83	7737.50 ± 4426.84	0.650
Day 1	7442.63 ± 3872.55	7208.87 ± 4102.91	0.908
Day 3	7392.63 ± 3393.37	6875.00 ± 3072.92	0.754
Mean	7191.33 ± 3954.00	7273.79 ± 3754.08	0.451
Lymphocyte, (800–5000/mL)			
Day 0	904.25 ± 413.41	1293.38 ± 540.79	0.128
Day 1	787.75 ± 390.15	1483.63 ± 725.82	0.320
Day 3	2332.25 ± 4240.02	1622.25 ± 458.28	0.754
Mean	1341.41 ± 534.90	1466.42 ± 576.39	0.267
Platelet, (150 000–40 000/mL)			
Day 0	201444.44 ± 111338.57	144375.00 ± 47847.65	0.200
Day 1	204111.11 ± 107639.96	127475.00 ± 67754.00	0.104
Day 3	153600.00 ± 113408.00	155625.00 ± 84971.06	0.964
Mean	186987.55 ± 108956.98	142491.67 ± 55027.36	0.352
Hemoglobin, (12.95–16.15 g/dL)			
Day 0	11.87 ± 2.92	11.59 ± 1.66	0.815
Day 1	11.26 ± 2.86	11.21 ± 1.63	0.971
Day 3	10.78 ± 3.02	10.98 ± 1.70	0.873
Mean	11.32 ± 2.75	11.26 ± 1.61	0.858
BUN, (7–20 mg/dL)			
Day 0	39.11 ± 20.45	22.25 ± 6.69	0.073
Day 1	40.78 ± 24.22	21.88 ± 5.61	0.069
Day 3	44.25 ± 25.60	18.63 ± 6.32	0.087
Mean	41.26 ± 23.45	20.92 ± 61.78	0.260
Creatinine, (0.65–1.2 mg/dL)			
Day 0	2.13 ± 2.20	1.00 ± 0.214	0.170
Day 1	2.28 ± 2.50	0.94 ± 0.17	0.153
Day 3	2.28 ± 2.50	0.93 ± 0.17	0.150
Mean	2.22 ± 2.39	0.95 ± 0.175	0.164
AST, (8–33 U/L)			
Day 0	61.11 ± 50.37	53.38 ± 21.63	0.694
Day 1	43.67 ± 30.19	53.88 ± 29.31	0.491
Day 3	41.50 ± 33.64	41.88 ± 21.78	0.979
Mean	49.03 ± 38.23	49.71 ± 24.09	0.890
ALT, (4–36 U/L)			
Day 0	32.67 ± 15.93	30.63 ± 9.05	0.754
Day 1	30.33 ± 14.58	31.75 ± 12.37	0.833
Day 3	29.87 ± 15.77	28.25 ± 17.18	0.847
Mean	30.99 ± 15.41	30.21 ± 12.79	0.986
LDH, (105–333 IU/L)			
Day 0	792.33 ± 539.10	670.25 ± 110.29	0.540
Day 1	710.59 ± 405.86	672.88 ± 133.79	0.806
Day 3	868.38 ± 452.11	602.00 ± 149.66	0.150
Mean	787.43 ± 466.21	648.38 ± 130.73	0.480
CPK, (10–120 μg/L)			
Day 0	226.50 ± 252.35	363.00 ± 470.11	0.481
Day 1	179.00 ± 133.17	256.25 ± 345.96	0.568
Day 3	197.00 ± 148.99	155.00 ± 228.20	0.671
Mean	199.96 ± 176.37	258.08 ± 356.46	0.715
Length of Hospitalization (day)	15.00 ± 10.13	17.89 ± 13.75	0.619
Medication to final outcome (day)	8.78 ± 7.74	13.78 ± 12.88	0.333
Medication torelease (day)	12.40 ± 9.01	15.17 ± 15.45	0.99<
Final outcome hospital discharge	5 (55.6%)	6 (66.7%)	0.99<
Death	5 (55.6%)	3 (33.3%)	0.592

Abbreviations: ALT, Alanine transaminase; AST, aspartate aminotransferase; CPK, Creatine phosphokinase; GCS, Glasgow Coma Scale; LDH, Lactate dehydrogenase.

## 4 DISCUSSION

To the best of our knowledge, this study represents the first randomized clinical trial aimed at investigating the potential effectiveness of modafinil in the treatment of patients suffering from COVID‐related encephalopathy. However, based on the results obtained, it is apparent that modafinil did not yield a statistically significant improvement in the level of consciousness among our patients. Specifically, the average Glasgow Coma Scale (GCS) score for patients in the treatment group exhibited a minor increase of 0.1, whereas in the placebo group, it decreased by 0.1. Nevertheless, when examining GCS scores at each measurement time point and the overall trajectory of the study, no significant differences were observed between the treatment and placebo groups.

We decided to administer modafinil and assess GCS scores every 2 h based on achieving a balance between modafinil's pharmacokinetic properties, notably its peak plasma concentration time, and the practical considerations of monitoring changes in patients' consciousness levels in a clinical setting. This approach enabled timely evaluations of modafinil's effectiveness while mitigating potential risks associated with delayed or infrequent assessments. Additionally, this method was preferred over a single 400 mg dose due to concerns about increased risk of side effects without clear short‐term benefits in consciousness levels.

We selected a one‐day standard administration period for modafinil based on its pharmacokinetics, with a half‐life of 12 to 15 h, ensuring therapeutic plasma levels without the need for multiple doses. By targeting the peak therapeutic window 2 to 4 h post‐administration, we aimed to assess modafinil's efficacy in enhancing consciousness promptly while minimizing accumulation risks associated with a longer treatment period.^9^ This approach allowed for timely evaluation with reduced adverse effects in a sensitive patient population. Moreover, Given the acute nature and rapid progression of COVID‐19 encephalopathy, swift therapeutic interventions are imperative for evaluating efficacy and tailoring treatment. A one‐day standard administration period for modafinil allows for immediate assessment of its potential to enhance consciousness levels. This critical timeframe enables prompt adjustments to treatment plans based on therapeutic response, optimizing patient outcomes amidst the rapidly evolving clinical scenario of COVID‐19 encephalopathy. Most importantly, our decision to restrict the initial administration period of modafinil to 1 day was driven by ethical concerns to minimize potential side effects, particularly in vulnerable patients with COVID‐19 encephalopathy. This cautious approach strikes a balance between the investigational nature of modafinil's use and our ethical obligation to prioritize patient safety and well‐being. Treatment extension is contemplated solely in cases exhibiting clear benefits, in accordance with principles of patient‐centered care.

We established that a one‐unit increase in the GCS score within 24 h of the initial dose would suggest modafinil's potential benefit on consciousness levels, based on literature indicating the clinical significance of such improvement, especially in acute neurological conditions.^8^, ^9^ If improvement was observed, modafinil administration continued for an additional 2 weeks at a daily dose of 400 mg, in line with effective dosing strategies in similar neurological applications.^9^, ^10^ Monitoring for side effects or adverse reactions was conducted to minimize unnecessary medication exposure, guided by ethical considerations and the principle of “do no harm.” These criteria were designed to personalize treatment based on individual responses to modafinil, enabling early identification of responders and non‐responders. Clear guidelines for continuation or discontinuation aimed to reduce subjective interpretation and enhance result reproducibility.

Regarding laboratory parameters, it is noteworthy that the level of creatinine in the treatment group was higher compared to the placebo group. This disparity can be attributed to the presence of two patients in the intervention group who were concurrently managing chronic kidney disease and were undergoing regular hemodialysis, thus explaining the observed difference. However, the variation in blood urea nitrogen (BUN) levels between the two groups did not reach statistical significance.

Among the numerous influential factors associated with the severity and mortality of COVID‐19, including advanced age and underlying medical conditions such as cardiovascular diseases, diabetes, hypertension, chronic lung diseases, chronic kidney diseases, malignancies, and laboratory abnormalities, no significant differences were observed between the two groups in our study. This absence of significant differences underscores the effectiveness of the randomization process employed in our study. It is noteworthy that the number of previously published studies addressing the topic of using modafinil in COVID‐19 patients with altered consciousness is limited. The majority of prior clinical trials primarily focused on evaluating the impact of modafinil on alertness, fatigue, and sleepiness. Only two studies, each with distinct designs, have thus far examined the effect of modafinil on improving consciousness in COVID‐19 patients.

One such study, a case report conducted by Roy et al.^13^ in 2021, investigated the use of modafinil in combination with Levodopa for treating a coma of unknown cause and hypokinetic rigid syndrome in an intubated 60‐year‐old patient with COVID‐19. According to this report, the patient was administered a daily dose of 200 mg of modafinil in the morning, along with 25 to 100 mg of Levodopa three times a day, for a duration of 1 month. Remarkably, this treatment regimen resulted in an improvement in consciousness, ultimately leading to the patient's successful separation from mechanical ventilation.^13^ Our randomized clinical trial examined the effects of modafinil on the level of consciousness among patients. However, our results did not reveal any significant impact of modafinil on consciousness levels. Unlike the single patient case discussed, our study subjects were not intubated, meaning they did not exhibit the same level of respiratory failure or severity of lung involvement. This difference in patient population may have contributed to the variance in outcomes observed between the case report and our trial. Additionally, the patient in the case report presented with a rigid syndrome necessitating the use of levodopa alongside modafinil. In contrast, our trial participants did not have a similar syndrome and therefore did not receive levodopa concurrently with modafinil. Notably, the administration protocols differed between the case report and our trial. The patient in the case report received a single daily dose of 200 mg of modafinil for 30 days, with noticeable changes in wakefulness and alertness within 2 days. In our trial, we administered modafinil at varying doses, starting from 100 mg every 2 h and reaching a peak dosage of 400 mg daily for 2 weeks. Our trial employed higher doses of modafinil administered at more frequent intervals compared to the case report. Despite this disparity, it is essential to acknowledge that both modafinil and levodopa, as mentioned in the case report, showed effects within 2 days of initiation, suggesting potential differences in responsiveness between individual patients and our study cohort. Furthermore, the case report mentioned specific brain pathologies, such as basal ganglia and corona radiata stroke observed in MRI imaging. In contrast, our trial lacked detailed information regarding the brain conditions of our participants, which could have influenced the observed outcomes.

In the study conducted by Amer et al.^14^ in 2021, the primary objective was to investigate the impact of modafinil on wakefulness in intubated patients who were hospitalized in the intensive care unit. Among the participants, 8 patients were administered daily doses of 100–200 mg of modafinil over a period of 4 days. This patient cohort consisted of individuals with various medical conditions, including 3 COVID‐19 cases, 4 surgical patients, and 1 transplant recipient. The study compared the GCS scores of these patients 48 h before commencing modafinil treatment with those obtained 168 h (1 week) after the initiation of drug administration. The study's findings indicated that GCS scores increased by 3–4 units in 5 patients, one of whom had a COVID‐19 diagnosis. Notably, the COVID‐19 patient who experienced a GCS increase was successfully extubated.^14^ It is crucial to note that the study being discussed was an observational case series, lacking a control group. Remarkably, only one out of three COVID‐19 patients exhibited a significant response to modafinil in terms of wakefulness. Unlike our randomized clinical trial, where patients were not intubated, all patients in the case series were intubated, potentially indicating more severe cases of lung involvement. In our trial, we administered higher doses of modafinil (ranging from 100 to 400 mg) over a longer duration (2 weeks) to nine patients, with another nine patients serving as the control group. However, there was no notable improvement in the GCS scores of the patients who received modafinil. Conversely, in the mentioned case series, one COVID‐19 patient showed increased GCS scores following modafinil administration. Notably, this patient had an ischemic stroke with hemorrhagic transformation in neuroimaging. Unfortunately, we lacked insights into the brain conditions of our patients. Consequently, the observed improvements in alertness and GCS scores in the case series cannot be conclusively attributed solely to modafinil. Various factors, including underlying conditions such as stroke, may have contributed to these changes. Given these discrepancies, further controlled studies are warranted to establish a definitive causal relationship between modafinil administration and improvements in wakefulness and GCS scores among COVID‐19 patients.

In a retrospective cohort study conducted by Mo et al., a total of 60 patients who were admitted to the intensive care unit and required mechanical ventilation for various reasons, including surgical, internal, and neurological conditions, were administered an average dose of 170 mg of modafinil over a 9‐day period. The findings from their study indicated that modafinil resulted in a transient and statistically non‐significant increase in the GCS scores of these patients.^15^ Both our RCT and this retrospective study yielded similar findings, indicating that modafinil wasn't effective in increasing patient wakefulness. This consistency in results strengthens the reliability of our findings and underscores the need to critically evaluate modafinil's efficacy in diverse patient populations. A significant disparity between the two studies lies in the patient populations. While our trial focused exclusively on COVID‐19 patients, the retrospective study included patients admitted to the ICU requiring mechanical ventilation. This variation in patient populations highlights the importance of considering disease‐specific factors and severity levels when interpreting outcomes. Patients requiring mechanical ventilation in the ICU typically represent a subset with more severe illness compared to COVID‐19 patients. The differing disease severity between the two cohorts may influence the response to modafinil, as the underlying pathophysiology and physiological states could vary significantly. Another notable difference is the dosage and duration of modafinil administration. While our trial utilized higher doses over a longer duration tailored specifically for COVID‐19 patients, the retrospective study employed lower doses for a slightly shorter period in a different patient population. Despite the retrospective study having a larger sample size, it is essential to consider the quality of data and potential biases inherent in retrospective analyses. The inclusion of a larger number of patients may enhance statistical power but does not inherently guarantee the reliability or generalizability of the results, particularly given the differences in patient populations and study designs. The consistent findings across both studies regarding modafinil's lack of efficacy in increasing wakefulness prompt careful interpretation. While the specific patient populations and dosing regimens differ, the congruence in outcomes suggests that modafinil may not be an effective intervention for improving wakefulness in critically ill patients, irrespective of the underlying condition.

In the randomized clinical trial conducted by Moradi Moghadam et al., the study focused on 60 patients who had sustained traumatic brain injuries (TBI) and had GCS scores ranging from 9 to 13. These patients were randomly allocated into either the intervention group or the placebo group. The individuals in the intervention group received a daily dose of 200 mg of modafinil for a period of 9 days. The GCS scores of the patients were subsequently reevaluated at 24 h and 196 h following the initiation of the treatment. The study's findings indicated that there was no significant difference in the level of consciousness observed between the two groups.^16^ Both our study and this RCT produced similar results, indicating that modafinil did not effectively increase patient wakefulness. This consistency suggests that modafinil may not be beneficial for improving wakefulness across various patient groups. The other RCT featured a larger population, further bolstering the robustness and generalizability of the results. However, a notable distinction between the two studies lies in the patient populations as our RCT focused on COVID‐19 patients, while the other RCT study involved individuals with traumatic brain injuries. Patients with traumatic brain injuries often face distinct neurological challenges and wakefulness disturbances compared to those with COVID‐19, potentially influencing their responsiveness to modafinil and contributing to differences in observed outcomes. Another significant contrast lies in the dosage and duration of modafinil administration. While our trial administered higher doses tailored for COVID‐19 patients over an extended period, the other study used lower doses for a slightly shorter duration in patients with traumatic brain injuries. These variations in dosing and duration may affect the drug's pharmacokinetics and therapeutic effects. The consistent results from both RCTs have important clinical implications. Healthcare providers should exercise caution when considering modafinil as a treatment option for increasing wakefulness in critically ill patients, irrespective of the underlying condition. Further research endeavors are warranted to explore alternative treatment strategies that may be more efficacious in improving patient outcomes in this population. The consistency of results between our RCT involving COVID‐19 patients and the other RCT involving patients with traumatic brain injuries underscores the need for cautious interpretation and consideration of patient characteristics and underlying conditions when evaluating modafinil's efficacy. These findings provide valuable insights into the limitations of modafinil as a wakefulness‐promoting agent in critically ill patients and highlight the importance of exploring alternative treatment strategies in this population.

Our investigation presents early evidence suggesting a slight increase in consciousness levels among COVID‐19 encephalopathy patients who received modafinil. Although not statistically significant, this observation emphasizes the potential of modafinil as a treatment option for enhancing cognitive function in this patient group. Modafinil's ability to increase dopamine, histamine, and orexin levels in the brain promotes wakefulness, while its effects on glutamate and GABA neurotransmission enhance cognitive function. Additionally, by inhibiting the reuptake of noradrenaline and serotonin, modafinil further contributes to its wakefulness and cognitive enhancement effects. These mechanisms underlie modafinil's influence on improving alertness and cognitive function in various neurological disorders. Importantly, the benefits of modafinil in enhancing wakefulness and cognition can lead to better patient outcomes and reduced healthcare burden by addressing issues such as fatigue, improving medication adherence, enabling better participation in rehabilitation, and potentially saving costs for healthcare systems.^8^, ^9^

While modafinil shows promise as a therapeutic option, it is crucial to assess its safety profile and associated risks. Our study meticulously monitored for adverse events, noting mostly mild side effects consistent with other modafinil contexts. In our study, only one patient in the modafinil group experienced a seizure after receiving the second dose of the drug. Notably, other potential side effects associated with modafinil, such as headache, nausea and vomiting, anxiety, agitation, high blood pressure, tachycardia, dizziness, and insomnia, which have been reported in various studies, were not observed in our patients. It is important to acknowledge that the assessment of some of these potential complications may have been limited due to the altered level of consciousness (LOC) exhibited by our patients. It is also important to bear in mind that when considering modafinil as a treatment for COVID‐19 encephalopathy, clinicians must balance the ethical duty to alleviate suffering with the principle of doing no harm. This decision requires informed consent, thorough patient and family counseling, and careful monitoring for side effects to ensure patient safety and well‐being.

In summary, based on our findings and observations, it can be concluded that modafinil appears to be a safe medication when used in patients with altered consciousness caused by COVID‐19‐related encephalopathy. Furthermore, modafinil may have the potential to reduce the duration of hospitalization and complications associated with pulmonary COVID‐19. However, further research and larger clinical trials are warranted to confirm these findings and establish the safety and efficacy of modafinil in this patient population.

## 5 LIMITATIONS AND FUTURE DIRECTIONS

As previously mentioned, our study included a relatively small number of patients, totaling 18 individuals, even though the initially calculated sample size was 32. Several factors contributed to this smaller sample size:

1. Decreased COVID‐19 Hospitalizations: A notable reduction in hospitalizations related to COVID‐19 occurred due to widespread vaccination efforts. This decline in the number of COVID‐19 patients eligible for the study naturally impacted the sample size.
2. Other Causes of Altered Consciousness: Our study focused on patients with altered consciousness due to COVID‐19‐related encephalopathy. However, there are various other potential causes of altered consciousness and encephalopathy unrelated to COVID‐19, which excluded certain patients from the study.
3. Intubation Criteria: Our study specifically targeted non‐intubated patients with altered consciousness caused by COVID‐19‐related encephalopathy. Consequently, individuals who required intubation as part of their medical management were not included.

It is worth noting that a similar situation regarding a smaller‐than‐anticipated sample size was observed in the study conducted by Poulsen et al., which aimed to assess the effects of modafinil on post‐stroke fatigue. In their study, the number of patients fell short of the calculated sample size due to specific inclusion criteria and the availability of eligible participants. Such variations in sample size are not uncommon in clinical research and can be influenced by various factors related to the study's design and patient population.^17^

In our study, extra paraclinical proceedings like brain MRI, electroencephalography (EEG), and cerebrospinal fluid (CSF) analysis were not accomplished. Moreover, due to the small sample size and the short period of modafinil administration, the effects of modafinil on improving consciousness may be underestimated.

Another key limitation we identified was the relatively short duration of our study. Although the one‐day standard administration period for modafinil enabled an acute assessment of its impact on consciousness levels, it limited our ability to observe long‐term outcomes and potential delayed effects. This limitation is important for interpreting the study's findings, particularly regarding the sustained efficacy and safety of modafinil over longer durations. We propose that future research should incorporate extended follow‐up periods to capture these longer term outcomes.

Our study population was constrained in terms of demographic diversity, predominantly comprising patients from a single geographic region with relatively homogeneous baseline characteristics. This limitation may impact the generalizability of our findings to broader populations with diverse demographic and clinical backgrounds. We emphasize the necessity for future studies to involve a more diverse patient cohort. This includes exploring the efficacy of modafinil across various age groups, ethnicities, and comorbid conditions to ensure broader applicability of the findings.

We recognize the possibility of biases in treatment implementation, despite our stringent blinding and randomization protocols. Factors such as unconscious bias among the research team or differential adherence to treatment among participants may have influenced the outcomes. Additionally, we suggest further strategies for future studies to minimize the impact of such biases, such as providing enhanced training for research personnel and employing advanced adherence monitoring techniques.

The thorough discussion of these limitations not only emphasizes the importance of careful interpretation of our findings but also lays the groundwork for future research directions. We emphasize the significance of addressing these limitations in subsequent studies, suggesting specific methodological enhancements such as conducting multicenter trials to improve demographic diversity, extending study durations to evaluate sustained effects, and implementing more robust mechanisms to mitigate biases.

Due to the preliminary nature of our findings, we support the need for additional research to conclusively determine the benefit–risk balance of modafinil in treating COVID‐19 encephalopathy. Subsequent studies, with expanded sample sizes, extended durations, and more diverse patient groups, are essential for validating our results and gaining a comprehensive understanding of modafinil's therapeutic efficacy and safety in this context.

## 6 CONCLUSION

The findings from our study indicate that modafinil led to a transient improvement in the level of consciousness among patients with COVID‐19‐related encephalopathy. However, it is important to note that this improvement was not statistically significant when compared to the control group. Given the limitations of our study, including the relatively small sample size, conducting additional randomized clinical trials with larger and more representative sample sizes is recommended. Furthermore, extending the duration of modafinil treatment in future studies may provide valuable insights into its potential benefits in the management of altered consciousness in COVID‐19 patients. Such studies can help elucidate whether modafinil's effects become more pronounced with longer term administration and whether it can lead to significant clinical improvements.

## AUTHOR CONTRIBUTIONS

M. N, R. Gh, H. M: substantial contributions to the conception or design of the work; or the acquisition, analysis, or interpretation of data for the work, or preparation of tables and figures. A. A, P. I, and M. N L: agreement to be accountable for all aspects of the work in ensuring that questions related to the accuracy or integrity of any part of the work are appropriately investigated and resolved. F. T K, M. R, N. Y: drafting the work or revising it critically for important intellectual content. O. M M, M. E: approval of the final version to be published. All authors reviewed the manuscript.

## FUNDING INFORMATION

This study has no financial support.

## CONFLICT OF INTEREST STATEMENT

The authors declare no conflict of interest.

## ETHICAL APPROVAL

Approval of the Research Protocol by an Institutional Reviewer Board: After obtaining approval from the ethics committee of the Iran University of Medical Sciences, all information was collected and analyzed confidentially and anonymously. The people present in the project adhered to all the principles of Helsinki ethics. Before starting the work, a full description of the research objectives and working methods was presented to the officials of the centers and all the research units in written and verbal form.

Informed Consent: Informed written consent regarding participating in this study is obtained from all the participants.

Registry and the Registration No. of the Study/Trial: The Ethics code regarding the present study is as follows: IR.IUMS.REC.1399,1056. The trial registration number (TRN) for this study is as follows: IRCT20170903036041N3 which was registered on 23/5/2021.

Animal Studies: N/A.

## CONSENT FOR PUBLICATION

Not applicable.

## Data Availability

We are unable to share our raw data due to confidentiality agreements and the sensitive nature of the data utilized in our study. These constraints prevent us from disclosing the dataset beyond what is already included in the manuscript. However, detailed descriptions of our methodology are available to enhance understanding and address inquiries.

## References

[1] McIntosh K , Hirsch M , Bloom A . Coronavirus disease 2019 (COVID‐19): epidemiology, virology, and prevention. Lancet Infect Dis. 2020;1:2019–2020.

[2] Azhideh A , Menbari‐Oskouie I , Yousefi‐Asl M . Neurological manifestation of COVID‐19: a literature review. Int Clin Neurosci J. 2020;7(4):164–170.

[3] Islam MA , Cavestro C , Alam SS , Kundu S , Kamal MA , Reza F . Encephalitis in patients with COVID‐19: a systematic evidence‐based analysis. Cells. 2022;11(16):2575. 36010650 10.3390/cells11162575 PMC9406394

[4] Koralnik IJ , Tyler KL . COVID‐19: a global threat to the nervous system. Ann Neurol. 2020;88(1):1–11. 32506549 10.1002/ana.25807 PMC7300753

[5] Fotuhi M , Mian A , Meysami S , Raji CA . Neurobiology of COVID‐19. J Alzheimers Dis. 2020;76(1):3–19. 32538857 10.3233/JAD-200581 PMC7660990

[6] Deviatkina N , Upasona R . The use of sedative medications, their prevalence, and their sedation of patients with disorders of consciousness, as well as the stimulating and sedative effects of alcohol. Scientific Collection *«InterConf+*», (27 (133)):243–250. 2022.

[7] Tang H , Zhu Q , Li W , Qin S , Gong Y , Wang H , et al. Neurophysiology and treatment of disorders of consciousness induced by traumatic brain injury: orexin signaling as a potential therapeutic target. Curr Pharm Des. 2019;25(39):4208–4220. 31663471 10.2174/1381612825666191029101830

[8] Duchêne A , Perier M , Zhao Y , Liu X , Thomasson J , Chauveau F , et al. Impact of astroglial connexins on modafinil pharmacological properties. Sleep. 2016;39(6):1283–1292. 27091533 10.5665/sleep.5854 PMC4863218

[9] Ballon JS , Feifel D . A systematic review of modafinil: potential clinical uses and mechanisms of action. J Clin Psychiatry. 2006;67(4):554–566. 16669720 10.4088/jcp.v67n0406

[10] Gagnon DJ , Leclerc AM , Riker RR , Brown CS , May T , Nocella K , et al. Amantadine and modafinil as neurostimulants during post‐stroke care: a systematic review. Neurocrit Care. 2020;33:283–297. 32394130 10.1007/s12028-020-00977-5

[11] Jha A , Weintraub A , Allshouse A , Morey C , Cusick C , Kittelson J , et al. A randomized trial of modafinil for the treatment of fatigue and excessive daytime sleepiness in individuals with chronic traumatic brain injury. J Head Trauma Rehabil. 2008;23(1):52–63. 18219235 10.1097/01.HTR.0000308721.77911.ea

[12] Kaiser PR , Valko P , Werth E , Thomann J , Meier J , Stocker R , et al. Modafinil ameliorates excessive daytime sleepiness after traumatic brain injury. Neurology. 2010;75(20):1780–1785. 21079179 10.1212/WNL.0b013e3181fd62a2

[13] Roy D , Song J , Awad N , Zamudio P . Treatment of unexplained coma and hypokinetic‐rigid syndrome in a patient with COVID‐19. BMJ Case Rep. 2021;14(3):e239781. 10.1136/bcr-2020-239781 PMC7929831 33653852

[14] Amer M , Bawazeer M , Butt AS , Dahhan TI , Kseibi E , Jamil MG . Modafinil for wakefulness in the critical care units: a literature review and case series including COVID‐19 patients at a tertiary care Saudi hospital. medRxiv. 2021;02.11.21250832.

[15] Mo Y , Thomas MC , Miano TA , Stemp LI , Bonacum JT , Hutchins K , et al. Effect of modafinil on cognitive function in intensive care unit patients: a retrospective cohort study. J Clin Pharmacol. 2018;58(2):152–157. 28858394 10.1002/jcph.1002

[16] Moradi Moghadam O , Nematollahi N , Shiri Malek Abad E , Hasani V , Tabibkhooei A , Sheikhvatan M , et al. Effect of modafinil administration on the level of consciousness in patients with brain injuries of moderate severity. Trauma Mon. 2019;24(1):1–5.

[17] Poulsen MB , Damgaard B , Zerahn B , Overgaard K , Rasmussen RS . Modafinil may alleviate poststroke fatigue: a randomized, placebo‐controlled, double‐blinded trial. Stroke. 2015;46(12):3470–3477. 26534969 10.1161/STROKEAHA.115.010860

